# Focused ultrasound enhances the anesthetic effects of topical lidocaine in rats

**DOI:** 10.1186/s12871-021-01381-y

**Published:** 2021-05-21

**Authors:** Hyun-Chul Kim, Wonhye Lee, Mark Böhlke, Kyungho Yoon, Seung-Schik Yoo

**Affiliations:** 1 Department of Radiology, Brigham and Women’s Hospital, Harvard Medical School, 75 Francis Street, 02115 Boston, MA USA; 2 School of Pharmacy, Massachusetts College of Pharmacy and Health Sciences University, Boston, MA USA; 3 Center for Healthcare Robotics, Korea Institute of Science and Technology, Seoul, Republic of Korea

**Keywords:** Anesthesia, ultrasound, plasma protein binding, α1-acid glycoprotein, EEG, somatosensory evoked potential

## Abstract

**Background:**

High-intensity ultrasound has been used to induce acoustic cavitation in the skin and subsequently enhances skin permeability to deliver hydrophobic topical medications including lidocaine. In contrast, instead of changing skin permeability, pulsed application of low-intensity focused ultrasound (FUS) has shown to non-invasively and temporarily disrupt drug-plasma protein binding, thus has potential to enhance the anesthetic effects of hydrophilic lidocaine hydrochloride through unbinding it from serum/interstitial α1-acid glycoprotein (AAG).

**Methods:**

FUS, operating at fundamental frequency of 500 kHz, was applied pulse-mode (55-ms pulse duration, 4-Hz pulse repetition frequency) at a spatial-peak pulse-average intensity of 5 W/cm^2^. *In vitro* equilibrium dialysis was performed to measure the unbound concentration of lidocaine (lidocaine hydrochloride) from dialysis cassettes, one located at the sonication focus and the other outside the sonication path, all immersed in phosphate-buffered saline solution containing both lidocaine (10 µg/mL) and human AAG (5 mg/mL). In subsequent animal experiments (Sprague-Dawley rats, *n* = 10), somatosensory evoked potential (SSEP), elicited by electrical stimulations to the unilateral hind leg, was measured under three experimental conditions—applications of FUS to the unilateral thigh area at the site of administered topical lidocaine, FUS only, and lidocaine only. Skin temperature was measured before and after sonication. Passive cavitation detection was also performed during sonication to evaluate the presence of FUS-induced cavitation.

**Results:**

Sonication increased the unbound lidocaine concentration (8.7 ± 3.3 %) from the dialysis cassette, compared to that measured outside the sonication path (*P* < 0.001). Application of FUS alone did not alter the SSEP while administration of lidocaine reduced its P23 component (i.e., a positive peak at 23 ms latency). The FUS combined with lidocaine resulted in a further reduction of the P23 component (in a range of 21.8 − 23.4 ms after the electrical stimulations; ***F***(2,27) = 3.2 − 4.0, *P* < 0.05), indicative of the enhanced anesthetic effect of the lidocaine. Administration of FUS neither induced cavitation nor altered skin conductance or temperature, suggesting that skin permeability was unaffected.

**Conclusions:**

Unbinding lidocaine from the plasma proteins by exposure to non-thermal low-intensity ultrasound is attributed as the main mechanism behind the observation.

## Background

Lidocaine acts on peripheral nerves by reversibly blocking sodium channels on the cell membrane, thereby inhibiting depolarization. Therefore, lidocaine has been used as local anesthetics and nerve blocking agents, as well as to treat ventricular tachycardia [1–3]. Cutaneous administration of hydrophilic lidocaine hydrochloride, available in various forms of delivery, is also widely used to provide temporary relief of muscle or peripheral pain. The stratum corneum of the skin only allows limited passage of hydrophilic molecules intracellularly by corneocytes, which is extremely limited [4]. Therefore, efficient transdermal transfer of these molecules is challenging in intact skin while only limited transfer may occur through discontinuous cutaneous structures, such as hair shafts or sweat glands [5]. Efforts have been made to improve its delivery; e.g., through the use of vehicles such as liposome [6], needleless gas-based transcutaneous injector [7], and iontophoresis by application of electrical currents to the skin [8].

Sonophoresis of the skin due to cavitation induced by ultrasound have also been used to increase skin permeability to drugs [9, 10], and have shown to be effective in reducing pain associated with venipuncture procedures in humans [11, 12]. However, the approach has uncertain long-term applicability due to its invasive nature (although minimal). Application of low-intensity ultrasound at intensities that do not raise tissue temperature or cause mechanical cavitation, has recently shown to unbind pharmacological agents from plasma proteins, and increase their transport to the adjacent tissue parenchyma [13]. The binding ratio of lidocaine to α1-acid glycoprotein (AAG), one of the major plasma proteins, is about 60 % [14, 15], and when bound, its pharmacological effects are reduced. The interesting utility of ultrasound in unbinding drugs from the plasma protein motivated us to examine the effects of low-intensity ultrasound, particularly at a level which does not yield cavitation, on increasing the unbound concentration of topically delivered hydrophilic lidocaine hydrochloride without altering the barrier function of the skin. The unbound lidocaine from the interstitial/plasma AAG would subsequently enhance its anesthetic effect.

Focused ultrasound (FUS) allows for localized delivery of acoustic energy to regional tissue while minimizing exposure to acoustic energy at the skin. Administration of quantifiable, low-intensity acoustic energy to region-specific tissue underneath the skin is especially conducive to examining the non-thermal and non-cavitational effects of ultrasound in unbinding lidocaine hydrochloride from serum/interstitial AAG.

## Methods

### Overview

We applied FUS, in a pulsed manner, to a saline solution containing physiologic concentration of lidocaine hydrochloride (referred to as ‘lidocaine’) and AAG, and measured unbound levels of lidocaine using equilibrium dialysis technique [16]. In a separate *in vivo* study, we measured the electroencephalogram (EEG) somatosensory evoked potential (SSEP) elicited by electrical stimulation of a unilateral hind leg of rats in order to characterize the anesthetic effects of topical lidocaine in nerve conduction. The SSEP was acquired from different experimental conditions—application of ultrasound to the thigh area following topical administration of lidocaine, ultrasound only, and lidocaine only. Since pulsed application of ultrasound itself has shown to modulate nerve conduction [17–19], we also examined the effects of FUS on SSEP without administration of lidocaine. To further probe changes in skin permeability, skin conductance and temperature were measured before, during, and after the application of FUS. Also, potential acoustic cavitation effects on the skin and underlying tissue were examined.

### FUS setup

**Fig. 1:**
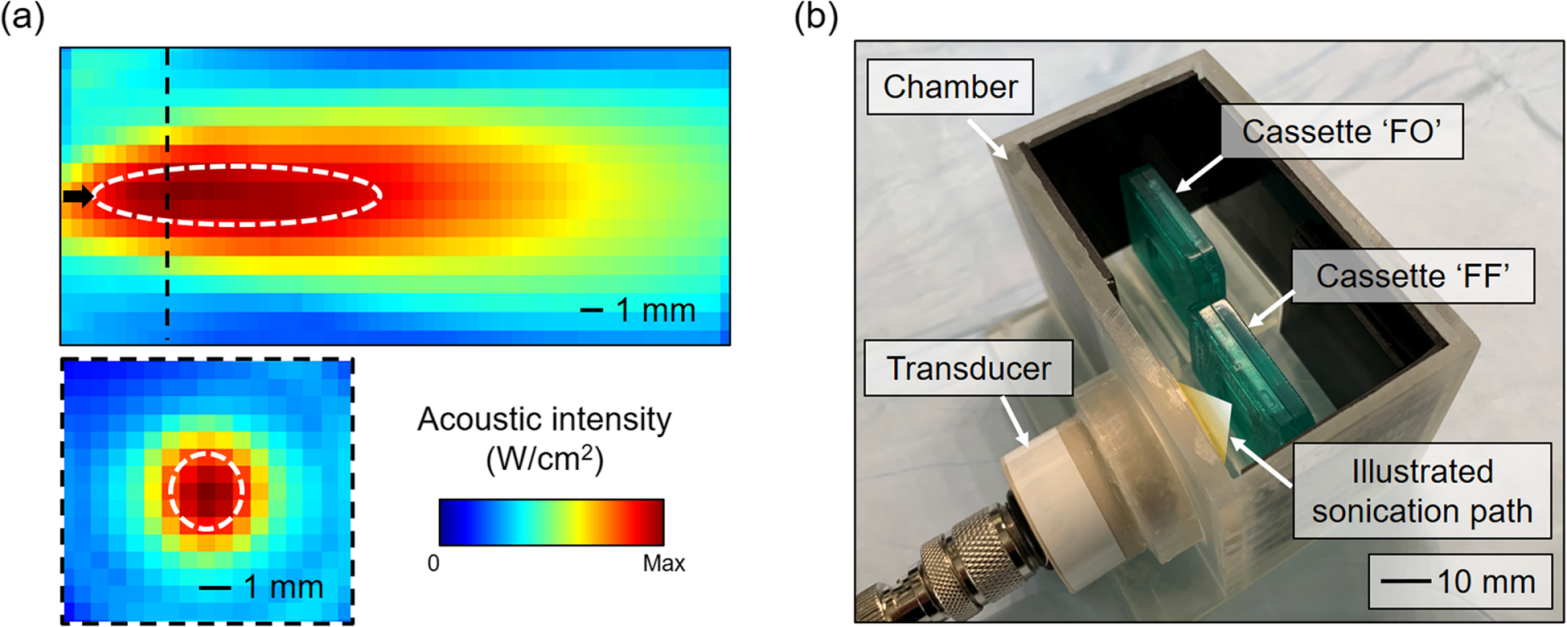
Acoustic intensity profile and equilibrium dialysis setup. **a** The acoustic intensity profiles across the longitudinal plane (top panel) measured 3 mm away from the exit plane of the transducer, and the transverse profile (bottom panel) at the focus indicated as a dotted black line (top panel). The full width at 90 %-maximum intensity is depicted with dotted white lines. **b** The experimental setting for the equilibrium dialysis, showing the dialysis chamber with rubber inserts (black pads). Dialysis cassettes at the focus (‘FF’) and outside the path of sonication (‘FO’) are shown with the illustrated sonication path

The acoustic intensity profile of the single-element FUS transducer (fundamental frequency, f_0_, of 500 kHz, GS500, Ultran Group, State College, PA, USA) was characterized using a needled-type hydrophone (HNC200, Onda, Sunnyvale, CA, USA) in degassed water. The detailed methods for the characterization process are described elsewhere [20]. The sinusoidal waveform signal to actuate the transducer was generated by a function generator (33500B, Keysight, Santa Rosa, CA, USA) and amplified by a linear power amplifier (Sonomo 500, Electronics and Innovations, Rochester, NY, USA). The acoustic focus formed 8 mm away from the exit plane of the transducer, whereby the ellipsoidal geometry of the acoustic focus was 2 mm in diameter and 12 mm in length (defined at the full width at 90 %-maximum intensity; dotted white line in Fig. 1a). FUS was given with 55-ms pulse duration and 4-Hz pulse repetition frequency at a spatial-peak pulse-average intensity (I_sppa_) of 5 W/cm^2^. These sonication parameters have shown to unbind phenytoin from the plasma protein, albumin at 250 kHz [13]. At the focus, the spatial-peak time-average intensity (I_spta_) was 1.1 W/cm^2^ while the corresponding peak negative pressure level was 384 kPa (corresponding mechanical index of 0.5). The mechanical index, a unitless number estimating the risk of mechanical damage from the sound waves (higher the number, higher the risk), is defined as the peak negative pressure level (in MPa) divided by the square root of fundamental frequency (in MHz). A mechanical index of 1.9 is the regulatory safety limit of the diagnostic ultrasound imaging devices [21], and thus the utilized pressure level, both in the sonication pathway and at the focus, was much lower than the level that may have any deleterious mechanical effects on biological tissue.

### Equilibrium dialysis of lidocaine and AAG

A chamber was built using 3-D printed components (Form 2, Formlabs, Somerville, MA, USA) to measure the *in vitro* effects of ultrasound on lidocaine-AAG binding (Fig. 1b). The chamber contained slots to hold the dialysis cassettes (7-kDa molecular weight cutoff pore size, Slide-A-Lyzer, Thermo Fisher Scientific, Waltham, MA, USA). 4 mm-thick rubber inserts padded the wall of the chamber to absorb acoustic waves. Lidocaine hydrochloride (PHR1257, MilliporeSigma, St. Louis, MO, USA) was dissolved in phosphate buffered saline (PBS, pH 7.4, Gibco 10,010, Thermo Fisher Scientific) at a concentration of 10 µg/mL (within the effective therapeutic range [22, 23]) having an AAG (G9885, MilliporeSigma) concentration of 5 mg/mL that simulates the maximum physiologic serum concentration in human [24, 25].

A 200 mL volume of the PBS-lidocaine-AAG solution was poured into the chamber and the 0.5 mL PBS without addition of lidocaine or AAG was injected to the dialysis cassettes after hydration. The cassette was placed 3 mm posterior from the location of the sonication focus (Fig. 1b, labeled ‘FF’) and the other outside the path of sonication (Fig. 1b, ‘FO’). A 7-kDa molecular weight cutoff pore size prevented the diffusion of human AAG (~ 42 kDa) into the dialysis cassettes while allowing the diffusion of unbound lidocaine (270.8 Da). The experiment was conducted at room temperature (~ 24 ºC), with the solution temperature equilibrated to the ambient temperature. The solution was not degassed, with an oxygen level at 6 ppm (measured using a dissolved oxygen assay kit, K-7512, CHEMetrics, Midland, VA, USA). Sonication was delivered for 1 h before retrieving the dialysates. The temperature of the solution was measured every 10 min using an infrared thermal camera (C3, FLIR Systems, Wilsonville, OR, USA) to ensure that there were no temperature-related confounders. The measurement was taken in 10 batches (each having ‘FF’ and ‘FO’ samples), half of which were prepared in freshly prepared solution.

Lidocaine concentrations were determined by liquid chromatography with tandem mass spectrometry (LC/MS/MS; HP1100 HPLC system, Agilent, Santa Clara, CA, USA) coupled to an AB/SCIEX 4000 triple quadrupole mass spectrometer (SCIEX, Framingham, MA, USA). A Capcell Pak MG-C18 column (1.5 × 50 mm, 3 μm particles, Phenomenex, Torrance, CA, USA) was used for separation, and the mobile phase was deionized water/acetonitrile (50:50 v/v) with 0.1 % formic acid added, pumped at a flow rate of 150 µL/min. Samples were prepared by adding 10 µL of sample to 800 µL acetonitrile containing 50 ng/mL lidocaine-d_10_ as an internal standard. These samples were further diluted by adding 50 µL the above mixture to 500 µL deionized water and 450 µL acetonitrile. Calibration standards were prepared by the same method. With the mass spectrometer in Multiple Reaction Monitoring mode, 5 µL injections were made and the transitions monitored were 235.3/86.3 for lidocaine and 245.3/96.3 for lidocaine-d_10_.

### Animal preparation and experimental setup

**Fig. 2:**
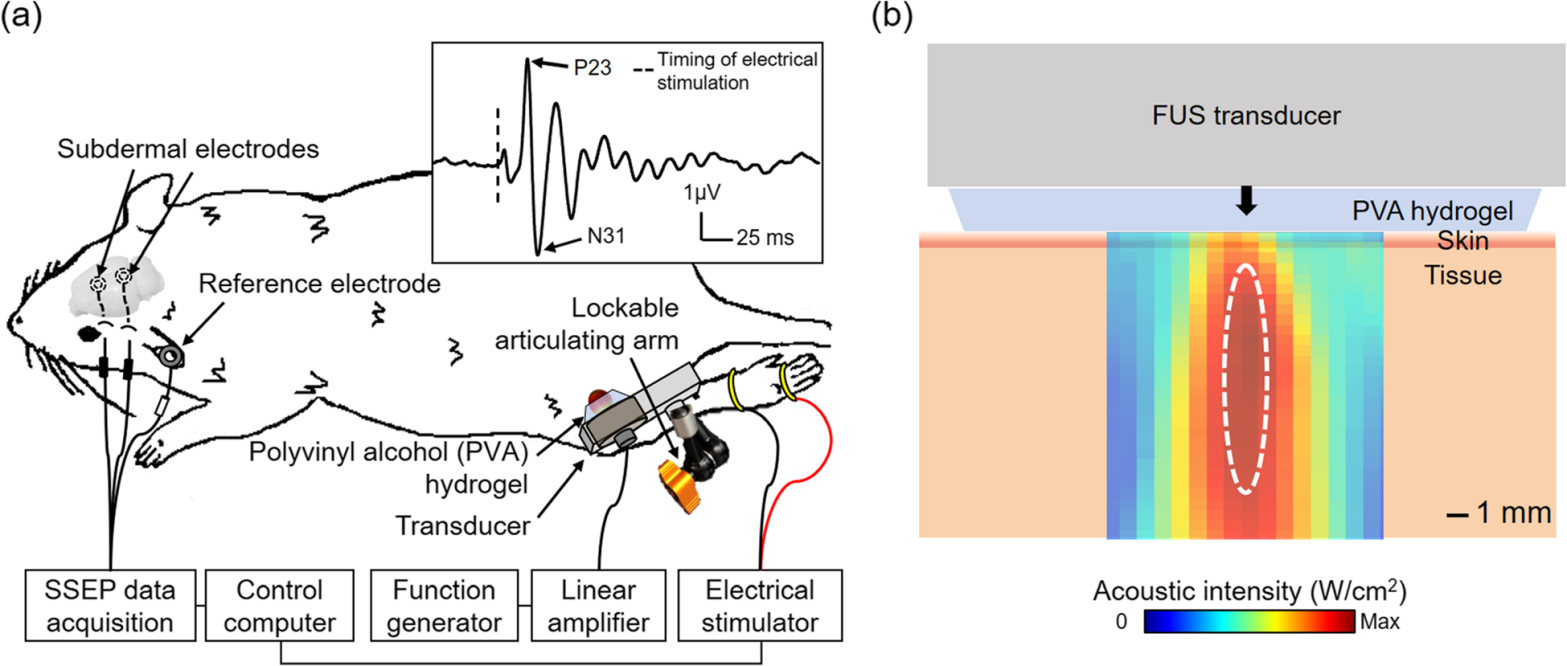
Rodent experimental setting for SSEP measurement and illustration of acoustic intensity profile at sonicated areas. **a** The schematics of the experimental setting with an example of an averaged SSEP (*n* = 10) elicited by electrical stimulation in the baseline time segment (‘US-/Lid-’, inset). **b** The illustration of the sonication to the tissue through the skin. The dotted white line indicates full width at 90 %-maximum acoustic intensity (refer to Fig. 1a for details of the acoustic spatial profile). The skin and tissue illustrations are not to scale

The study was conducted under the approval and according to guidelines and regulations set forth by Institutional Animal Care and Use Committee of Brigham and Women’s Hospital (Protocol #: 2020N000107), Boston, MA, USA. Sprague-Dawley rats (all males, *n* = 10) were anesthetized with intraperitoneal injection of ketamine/xylazine (80:10 mg/kg), and the furs over the scalp, hind legs, paws were removed using an electric clipper and a depilatory cream. The rats were then placed on a plastic platform in the prone position, and two subdermal EEG electrodes (SWE-L-25; Ives EEG Solution, Newburyport, MA, USA) were inserted under the scalp ~ 4 mm and ~ 13 mm rostral to the lambda to measure SSEP, with a AgCl cup electrode placed on the unilateral ear as a ground. An interaural line was used to estimate the location of the lambda. Band electrodes placed onto a hind paw (unilateral) were used to elicit the SSEP *via* an electrical stimulator (MLADDF30; ADInstruments, Colorado Springs, CO, USA) with application of conductive gel (g.GAMMAgel, g.tec, Schiedlberg, Austria) on the skin (illustrated in Fig. 2a).

A lockable articulating arm (MA207, Ikan, Houston, TX, USA) mounted to the platform was connected to a single-element FUS transducer and was used to hold the transducer in a prescribed position and orientation. The FUS transducer was placed over the thigh, aiming at the approximate location of the sciatic nerve. A 2mm-thick polyvinyl alcohol (PVA) hydrogel (341,584, MilliporeSigma; 9 % weight per volume in degassed water, two freeze-thaw cycles) was placed between the transducer and the skin for acoustic coupling [26], and allowed for placing acoustic focus ~ 6 mm under the skin (illustrated in Fig. 2b). Ultrasound gel (Aquasonic, Parker Laboratories, Fairfield, NJ, USA) was applied between PVA hydrogel and transducer surface. The zone encompassing the sciatic nerve in the thigh was chosen over the distal sural and saphenous nerves (which directly innervate the hind paw [27]) due to its proximity to the brain and ease of anatomy-based identification/sonication. Except for the time needed for the application of the topical lidocaine, the transducer remained in position throughout the SSEP measurement.

SSEP was acquired at a sampling rate of 10 kHz in a time-locked fashion covering 50 ms before and 250 ms after the onset of the electrical stimulations (constant current of 9 mA, 50 µs duration) that were delivered to the unilateral hind paw area 500 times at a 2 Hz rate. SSEP from each electrical stimulation event was filtered with Mains filter, notch filter (60 Hz), and band-pass filter (0.8 − 100 Hz), and was subjected to linear detrending and baseline-correction (with respect to -50 − 0 ms). The SSEP averaged across the animals from ‘US-/Lid-’ showed distinctive positive and negative peaks at 23-ms (P23) and 31-ms (N31) latencies, respectively (inset in Fig. 2a).

**Fig. 3:**
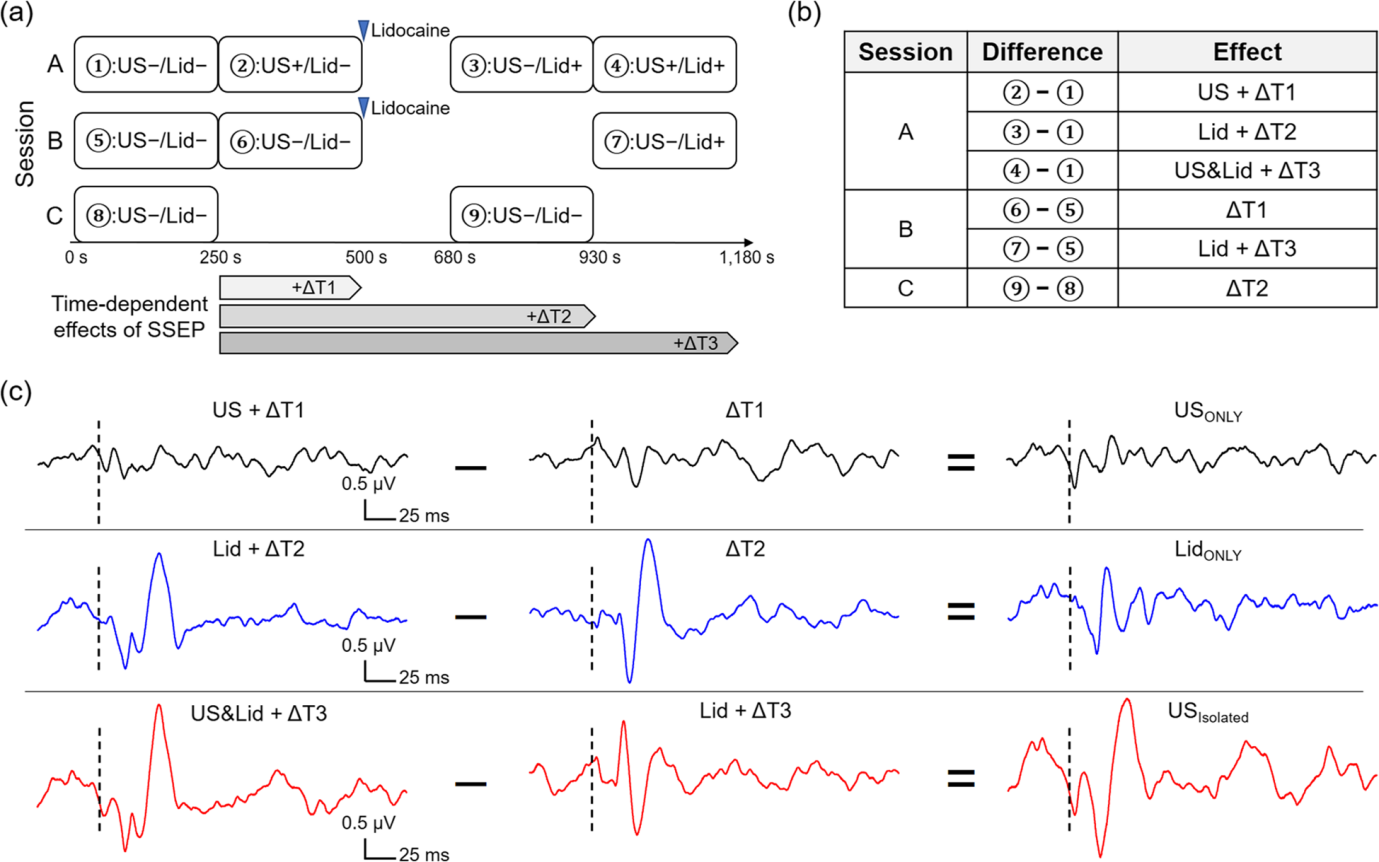
Data acquisition timeline and derivation of condition-specific SSEP. **a** Timeline of three sets of experiment sessions. The inverted blue arrowheads indicate the timing of lidocaine application. (US+/-, with/without ultrasound; Lid+/-, with/without lidocaine). ΔT*i* denotes time-dependent effects of SSEP from the (*i +* 1)^th^ segment (*i =* {1, 2, 3}) from each session. **b** The summary table on the derivation of SSEP features that contain both condition-specific and time-dependent effects within each session. **c** Group-averaged (*n* = 10) derivation of condition-specific SSEP (in the right column) by subtracting the SSEP data (e.g., the difference in SSEP segments with respect to the initial segment, ‘US-/Lid-’) of session B/C (middle column) from that of session A (left column). US_ONLY_ indicates the effect of ultrasound whereas Lid_ONLY_ indicates the effect of application of lidocaine. The effect of ultrasound after the application of lidocaine, being isolated from the application of lidocaine alone, is denoted as US_Isolated_. The dotted black lines indicate the timing of electrical stimulation

The time-variant depth of the anesthesia by intraperitoneal injection of ketamine/xylazine is known to alter SSEP [28]. To reduce its confounding effects on SSEP, three separate sets of SSEP sessions (labeled ‘A’ through ‘C’) were conducted with at least a three-day gap for full recovery from anesthesia (schematics shown in Fig. 3a). The side (left/right hind paw) of the electrical stimulation and the sequence of sessions were randomized and balanced. Session A consisted of sequential acquisition of SSEP segments (each 4 min and 10 s) under (1) no intervention (‘US-/Lid-’), (2) FUS only (‘US+/Lid-’), (3) lidocaine only (‘US-/Lid+’), and (4) FUS and lidocaine (‘US+/Lid+’). Session B consisted of two sequential SSEP acquisitions under no intervention (‘US-/Lid-‘) and SSEP acquisition without FUS after the administration of lidocaine (‘US-/Lid+’) acquired with a time gap (7 min 10 s). Session C consisted of two sets of measurements of SSEP without any intervention (‘US-/Lid-‘) that are separated by an appropriate time gap (7 min 10 s). The effects of the time gap among segments, including the time of SSEP acquisition, were considered independent to each other, and were represented as ΔT1 ~ ΔT3 in Fig. 3a. For acquisition of SSEP under the influence of lidocaine, 3 mL lidocaine cream (dispensed from a 10 mL syringe; 5 %, Advanced Numb, UberScientific, Lexington, KY, USA) was manually applied over the skin for ~ 5 seconds while the duration of application was not strictly controlled. The lidocaine cream did not have other additional elements that affect transport and protein binding. We did not apply additional ultrasound gel on the surface of the PVA coupler where it meets the skin to avoid dilution of the lidocaine cream. SSEP was measured 3 min after the application of lidocaine cream to a unilateral thigh area, considering the onset of adequate local anesthetic action of lidocaine (< 2 − 3 min) [29, 30].

The SSEP obtained during the initial segment was subtracted from the subsequent segment within the same session to isolate the signal features that contain both condition-specific and time-dependent effects (labeled in Fig. 3b). After deriving SSEP having the signal features that contain both condition-specific and time-dependent effects within each session, their differences were obtained (an example shown in Fig. 3c) to evaluate the effects of ultrasound (US_ONLY_) or lidocaine (Lid_ONLY_) separately, and to isolate the effects of ultrasound after the application of lidocaine (US_Isolated_). The negative values indicate the amount of reduction in SSEP amplitude for each condition.

The respiratory rates (breaths per min [bpm]), as an important indicator of anesthetic depth [31], were measured by visual counting for the duration of 30 s every five minutes. Skin conductance was monitored for 2 min, derived from galvanic skin response (GSR) using two electrodes (ML116, ADInstruments) placed on the skin with a 4-cm gap on the lateral side of the transducer before, during, and after the application of FUS. Skin temperature was also measured before and after the sonication using an infrared thermal camera (C3, thermal sensitivity < 0.1 °C and sensor resolution of 80 × 60 with 41° × 31° field-of-view, FLIR Systems, Wilsonville, OR, USA). The camera was calibrated on the black non-reflecting plastic block over the range of 30–40 °C (measured with thermistor-based thermometry) prior to use. Upon measurement and recovery from the anesthesia, the animals were returned to a vivarium and monitored for at least three days for the presence of any abnormal behavior.

### Evaluation of thermal effects

In addition to temperature measurement from the skin, the potential of thermal effects at the skin and the sonicated tissue area were estimated by sequentially solving the Khokhlov-Zabolotskaya-Kuznetsov and bio-heat transfer equations [32] through an open-source high intensity ultrasound simulator based on MATLAB scripts (https://www.fda.gov/about-fda/cdrh-offices/hitu-simulator) [33]. The estimation was performed using the maximum *in situ* acoustic intensity at the focus 5 W/cm^2^ I_sppa_ and at the skin 2.53 W/cm^2^ I_sppa_. The simulation was performed at resolution of 0.5 mm with a temporal resolution of 0.2 ms, using the acoustic properties (speed of sound of 1500 m/s, density of 1000 kg/m^3^) and thermal properties of the muscle (specific heat of 3465 J/kg·K^− 1^, thermal conductivity of 0.5 W/m·K^− 1^, perfusion rate of 0.009 kg/m^3^·s^− 1^) [34]. For the simulation of thermal effects at the skin, specific heat of 3300 J/kg·K^− 1^, thermal conductivity of 0.45 W/m·K^− 1^, and perfusion rate of 0.0013 kg/m^3^·s^− 1^ were used, with initial temperature of 37.5 ºC.

### Evaluation of cavitation

A passive cavitation detection technique was used to examine the presence of any cavitation by measuring the acoustic emission spectra associated with sonication [35, 36]. To do so, a broadband ultrasound transducer/detector (center frequency 1 MHz, full-width at half maximum detection range ~ 0.5–1.5 MHz, V303-SU, Olympus NDT, Waltham, MA) was applied next to the sonicated leg area of a rat, and emitted sound waves from the tissue were synchronously measured at the sonication onset (sweep time = 67.5 ms). As cavitation-related emissions are detected in a frequency range much higher than the applied ultrasound frequency (f_0_ = 500 kHz), i.e., in ultra-harmonic frequency components of 1.5 f_0_ (750 kHz), 2.5 f_0_ (1.25 MHz), and 3.5 f_0,_ (1.75 MHz) [36], the frequency spectrum of the detected signal from the transducer was obtained across 0–2 MHz range with a step size of 10 Hz at 20 MHz sampling rate using a spectrum analyzer (SSA3021X-TG Digital Spectrum Analyzer, Siglent, Transcat, Rochester, NY). To account for the frequency-dependent transducer sensitivity, the experiment was conducted with and without the sonication (*n* = 100 each), and the averaged spectra were subsequently compared through subtraction in frequency domain.

## Results

**Fig. 4:**
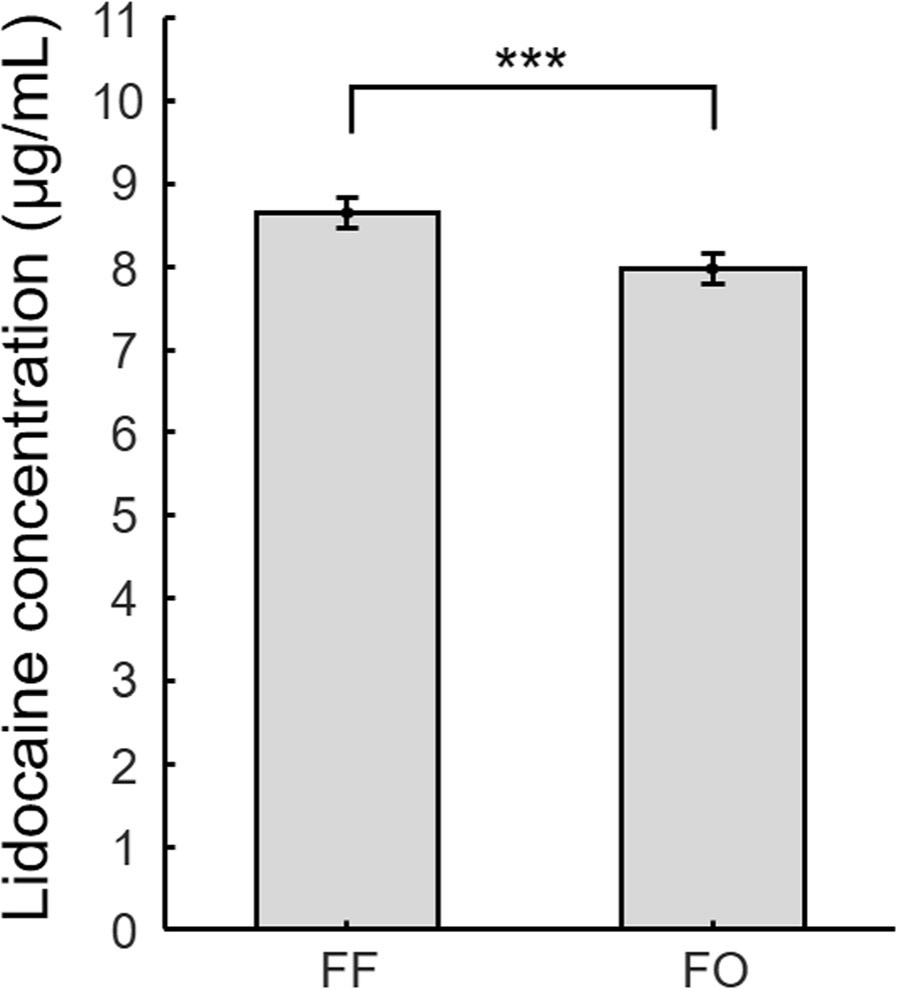
Comparison of lidocaine concentration in samples obtained at the sonication focus (FF) and outside the focus (FO). The bar and error bars indicate average values and standard deviations. The *P*-value was obtained from a one-tailed paired *t*-test. ^***^, *P* < 0.001

As depicted in Fig. 4, the concentration of lidocaine obtained using equilibrium dialysis was significantly elevated (8.7 ± 3.3 %, mean ± standard deviation) at the sonication site (8.66 ± 0.18 µg/mL) compared to the samples obtained outside of focus (7.97 ± 0.18 µg/mL; paired *t*-test, one-tail, *P* < 0.001). During the dialysis, the temperature did not change with time (24.39 ± 0.06 °C across 7 time points; one-way repeated measures analysis of variance [ANOVA], ***F***(6,54) = 1.64, *P* = 0.16) and across 10 batches of samples (one-way ANOVA, ***F***(6,63) = 0.69, *P* = 0.66).

In animal experiments, there was no difference in skin conductance (ΔGSR) before, during, and after FUS sonication (one-way repeated measures ANOVA, ***F***(2,18) = 1.2, *P* = 0.31; -1.5 ± 2.9, -0.8 ± 1.5, and − 1.4 ± 2.2 µS). FUS sonication did not change skin temperature (paired *t*-test, *P* = 0.30; 30.3 ± 3.1 °C before, and 30.0 ± 2.5 °C after sonication). These results indicate that the application of FUS did not alter either the skin permeability or temperature. The respiratory rate, which is one of primary indicators for the anesthetic depth, did not change within sessions (session A, one-way repeated measures ANOVA, ***F***(3,27) = 2.0, *P* = 0.13; 60.0 ± 5.0, 59.6 ± 5.1, 58.8 ± 5.7, and 58.4 ± 5.7 bpm in the sequential acquisitions; session B, ***F***(2,18) = 0.9, *P* = 0.44; 58.6 ± 4.4, 57.6 ± 2.8, and 57.2 ± 2.7 bpm; session C, paired *t*-test, *P* = 0.34; 59.6 ± 7.3 and 58.6 ± 5.4 bpm) and among the sessions (one-way ANOVA, ***F***(2,29) = 0.3, *P* = 0.78; 59.2 ± 5.2, 57.8 ± 2.7, and 59.1 ± 6.2 bpm, A through C). All animals showed normal behavior with no signs of skin damage during the post-sonication monitoring periods.

**Fig. 5:**
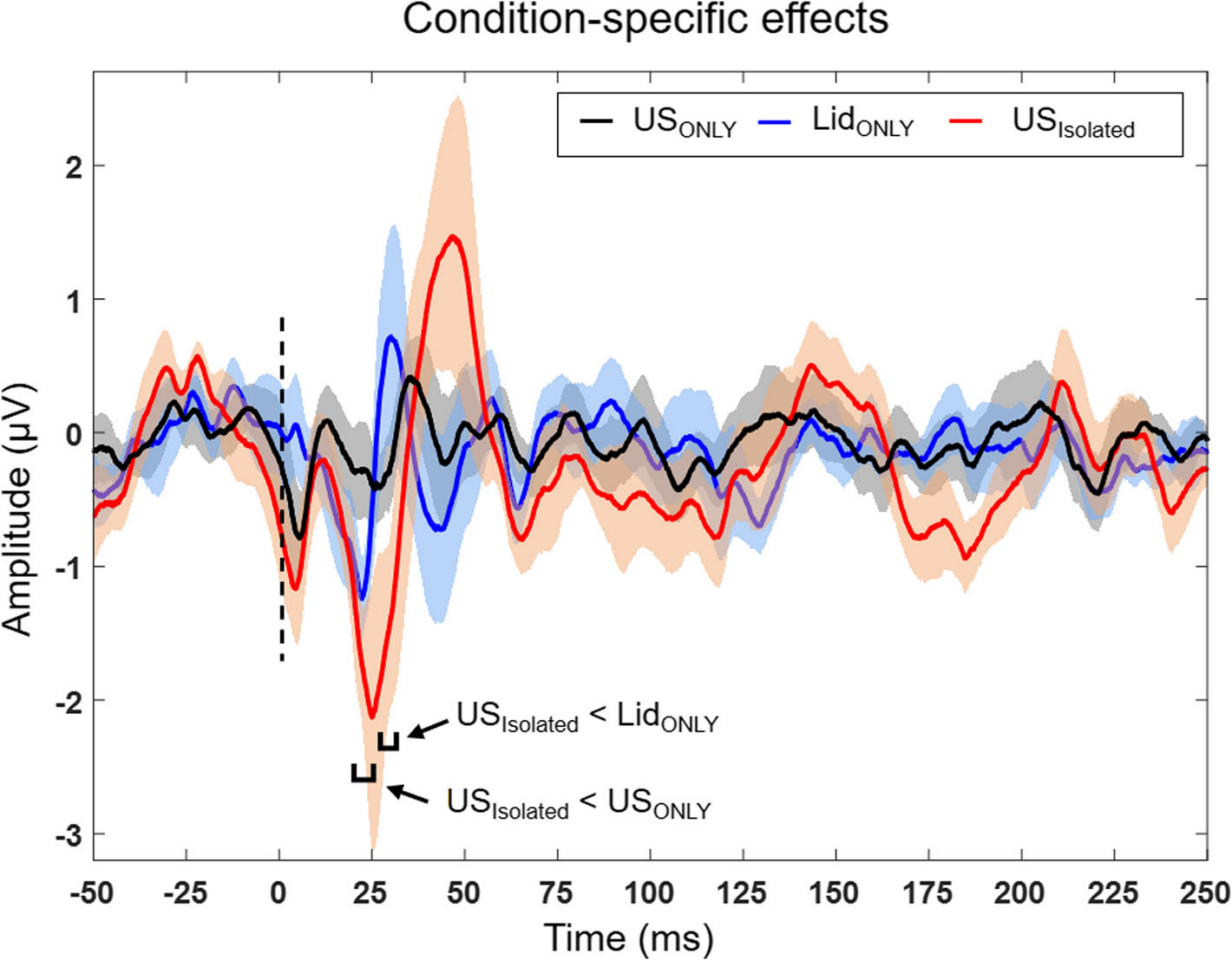
Condition-specific group-averaged SSEP. The subtraction procedure yielded condition-specific SSEP shown with the shades indicating standard errors. The brackets indicate the time segment showing differential signal features between the conditions (one-way ANOVA followed by Tukey-Kramer post-hoc analysis, *P* < 0.05). The dotted black lines indicate the timing of electrical stimulation

The condition-specific data were compared using one-way ANOVA followed by Tukey-Kramer post-hoc analysis. SSEP representing the effects of US_ONLY_ (in black line; Fig. 5) did not show any distinct peaks. In the Lid_ONLY_ condition, the P23 component slightly reduced (-0.9 ± 0.8 µV; in blue line; Fig. 5) compared to that of the US_ONLY_ condition; albeit without statistical significance (*P* = 0.18). The US_Isolated_ condition reduced the P23 component further (-1.8 ± 1.7 µV; in red line; Fig. 5) compared to the use of US_ONLY_ (-0.3 ± 1.1 µV), with statistical significance shown after 21.8 − 23.4 ms upon the electrical stimulations (Tukey-Kramer post-hoc analysis, ***F***(2,27) = 3.2 − 4.0, *P* < 0.05). The SSEP amplitude in the US_Isolated_ condition was also reduced in 26.9 − 30.2 ms after the stimulation, compared to that in the Lid_ONLY_ condition (Tukey-Kramer post-hoc analysis, ***F***(2,27) = 3.2 − 3.3, *P* < 0.05).

**Fig. 6:**
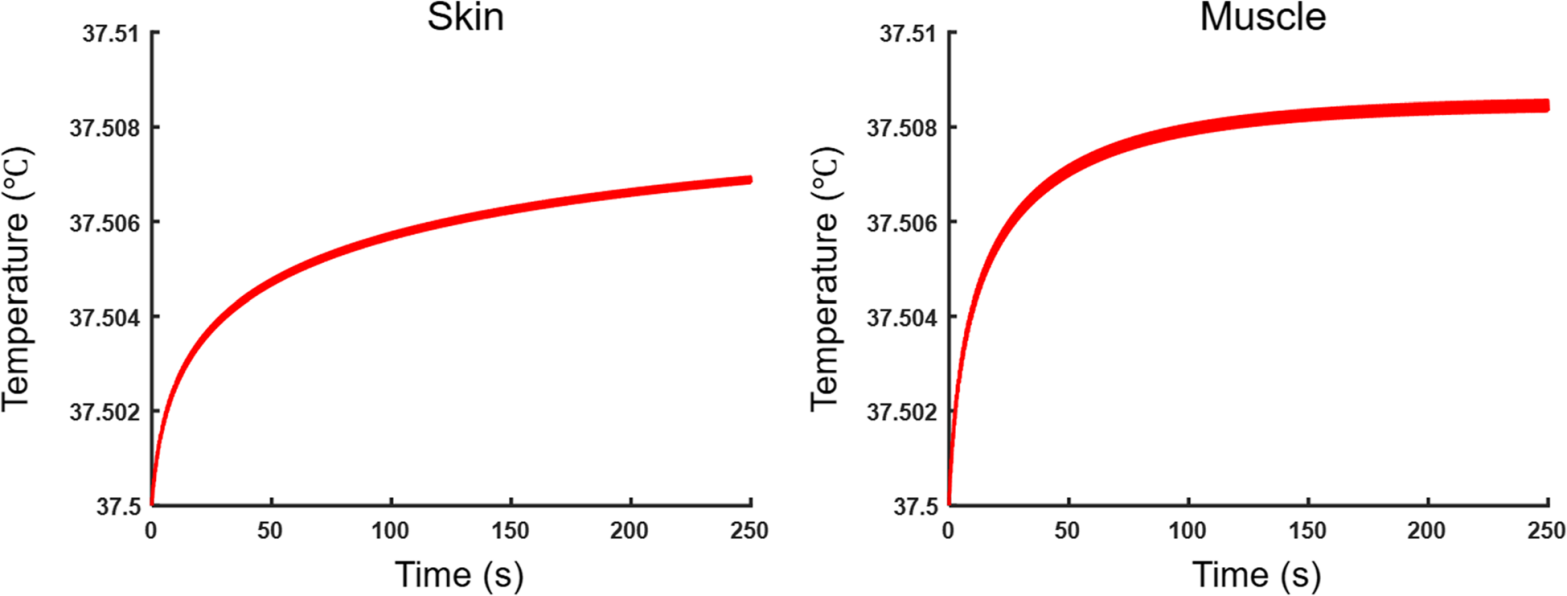
Estimated temperature rise at the sonicated skin and adjacent muscle through numerical simulation. The same set of sonication parameters adopted in the actual experiment was used with I_sppa_ = 2.53 W/cm^2^ on the skin and I_sppa_ = 5 W/cm^2^ on adjacent muscle

**Fig. 7:**
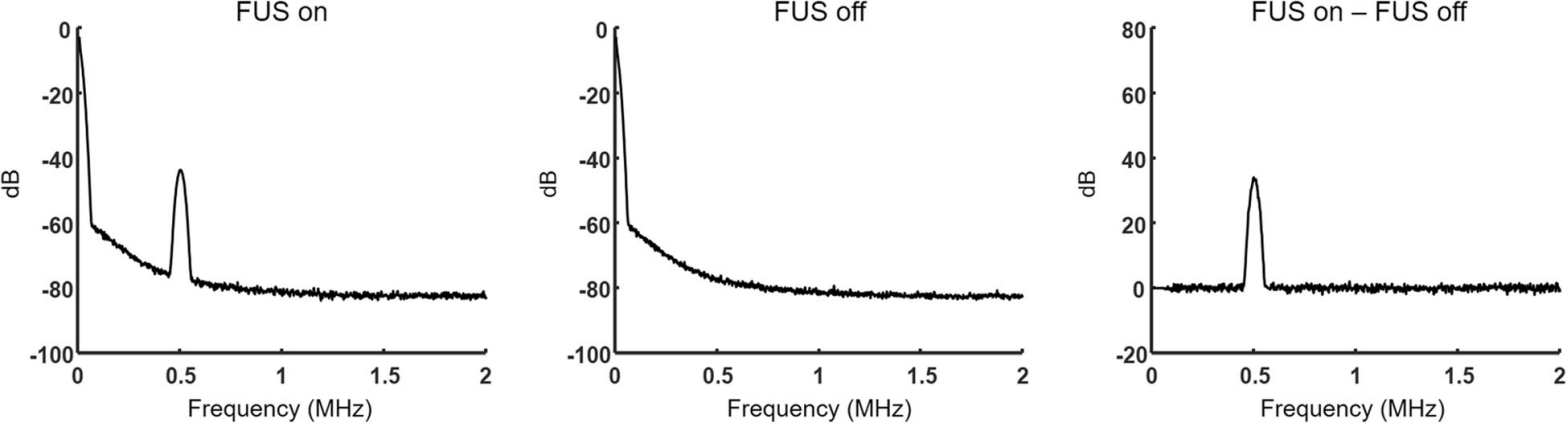
Frequency spectra of passive cavitation detection signals. Averaged frequency spectra of passive cavitation detection signals (*n* = 100) were obtained during sonication (FUS on; left panel), without sonication (FUS off; middle panel), and their spectra were compared by subtraction (right panel)

The thermal simulation results (Fig. 6) revealed negligible temperature rise in the muscle as well as at the skin (≤ 0.009 °C), confirming the temperature measurement at the skin. From examination of emission spectra associated with sonication (Fig. 7), a single peak at 500 kHz was detected, corresponding to the applied ultrasound frequency. No other spectral peaks were detected in ultra-harmonic frequency components, signifying the absence of cavitation.

## Discussion

Equilibrium dialysis of the lidocaine from AAG-lidocaine PBS solution showed an elevated lidocaine concentration in the sonicated region compared to that outside of the acoustic focus. The sonication parameters used in the present study, except for the use of higher frequency (500 kHz versus previous use of 250 kHz), have shown to be effective in unbinding phenytoin from plasma protein albumin [13], and the results provided evidence that a portion of the lidocaine was unbound from AAG by the application of ultrasound and diffused into the dialysis cassettes. As the forces that govern the drug–plasma protein binding are extremely weak (on the order of piconewton 10^− 12^ N), recognized as noncovalent electrostatic or Van der Waals forces [37], the results provided a rationale that pulsed application of ultrasound may also be used to unbind basic drugs, such as lidocaine, from the AAG in plasma.

The SSEP responding to the electrical stimulation of a hind paw was used as an indicator to evaluate the anesthetic effects of lidocaine in the *in vivo* portion of the study. Constant respiratory rates during the SSEP acquisition indicate that all animals were under the stable anesthetic plane. The time-dependent variations of SSEP under ketamine/xylazine anesthesia were compensated by subtracting the SSEP data obtained from the same timeline in a relatively short data acquisition duration of < 20 min. Application of ultrasound alone without the use of lidocaine did not affect the SSEP amplitude while topical application of lidocaine slightly reduced the P23 component. When ultrasound was applied to the area that was exposed to the topical lidocaine, the amplitude of the P23 component was decreased further, suggesting the enhanced anesthetic effect of lidocaine (Fig. 5).

The potential role of FUS in enhancing the effects of lidocaine can be explored in terms of its intravenous administration for epilepsy [38] or its injection to achieve nerve block [39]. As pain perception involves more complicated neural processing than the modulation of afferent signal to the brain [40], behavioral studies, such as administration of the technique among awake animals that are subjected to noxious stimulation, can help elucidate the ultimate effects of ultrasound on enhancing the anesthetic effects.

As to the potential mechanism behind our finding, the possibility of changes in skin permeability by ultrasound was ruled out since skin conductance was unaffected. Low-intensity ultrasound used in this work (mechanical index = 0.5) was far below the intensity compatible with ultrasound imaging applications (i.e., mechanical index = 1.9 [21]), and did not induce cavitation (Fig. 7) and associated change in skin permeability as measured by skin conductance. The thermal contribution was also unlikely due to the use of low intensity (I_spta_ at the focus was 1.1 W/cm^2^) that was well below the threshold of temperature change as supported by temperature measurement from the skin and through numerical thermal simulation (Fig. 6). We, however, acknowledge the absence of real-time monitoring of temperature from the sonicated tissue, and non-invasive temperature measurement technique, such as magnetic resonance thermometry [41], is needed to monitor the temperature *in situ* in future investigations.

Supported by *in vitro* equilibrium dialysis results, we conjecture that the observed enhancement of anesthetic effects was mediated by unbinding of lidocaine from the plasma proteins by acoustic radiation force, which accordingly increased the level of ‘free’ lidocaine that enhances its pharmacological action. Direct derivation of the acoustic radiation force at AAG-lidocaine complex is difficult since absorption coefficient of AAG was unknown. Instead, qualitative estimation was performed herein. Based on the dimension of AAG (~ 30 × 30 × 60 Å [7]), the maximum surface area exposed to the incident pressure wave was estimated 18 × 10^− 18^ m^2^ (30 Å × 60 Å estimated from rectangular geometry). Assuming full absorption of the pressure waves (0.38 MPa, i.e., 3.8 × 10^5^ N/m^2^ ) at the surface, the radiation force imposed on AAG approximates to 6.8 × 10^− 12^ N (Pressure × Area; 3.8 × 10^5^ N/m^2^ × 18 × 10^− 18^ m^2^), which is comparable to the binding force. Therefore, acoustic radiation force offers a plausible explanation for the observed phenomena. However, since the exact types and magnitude of binding forces that governs AAG-lidocaine interactions are not known, along with difficulty in making direct comparisons in terms of degree of unbinding between *in vitro* and *in vivo* settings, further investigation is needed to reveal quantifiable dynamics of ultrasound-mediated plasma protein-drug unbinding.

We note that the normal serum range of AAG in Sprague-Dawley rats is 0.23 − 0.32 mg/mL, which is significantly lower than that of humans [25, 42]. It is also important to understand that the concentration of plasma proteins may differ in interstitial fluid compared to the serum, for example, cutaneous interstitial fluid albumin concentration is about 62 % of the serum albumin in humans [43], whereas the AAG level may double in inflammatory conditions and during wound-healing [44, 45]. Therefore, it is reasonable to anticipate that a degree of plasma protein binding with lidocaine would vary among species, tissue macroenvironment, and specific pathological conditions. We also note that lidocaine concentration in the blood, either central or peripheral, was not measured in the present study, thus the level of circulating drug concentration was not known for each animal. As the drug concentration may vary significantly, being influenced by many factors (e.g., skin hydration, hair removal, and skin age), measurement of drug concentration from both blood and interstitial space warrants further investigation.

Selective unbinding of drugs from plasma proteins casts interesting possibilities beyond enhancing anesthetic effects as many types of drugs bind to plasma proteins (mainly to serum albumin in the cases of acidic/neutral drugs or to AAG in the case of basic drugs [46]). For example, ultrasound can be used to unbind anti-tumor drugs such as paclitaxel from the albumin (> 90 % binding to albumin) to enhance its delivery to a tumor region without increasing systemic dose. In this context, additional caution is advised to avoid cavitation-related damage when sonication needs to be delivered to proximity to organs that contain the gas (e.g., the lungs) as gas-containing tissues are more susceptible to cavitation [47]. A further study is warranted to examine the range of sonication parameters and their effects on unbinding various types of pharmacological agents from plasma proteins.

## Conclusions

We have found that pulsed-mode application of low-intensity FUS can unbind lidocaine from the AAG, which enhances its pharmacological action without thermal/cavitational effects of ultrasound. Focused administration of acoustic energy is especially conducive to increasing the availability of an unbound drug to a region-specific area located underneath the skin. The assessment of ultrasound pulsing schemes in achieving unbinding of a drug with the purpose of enhancing its delivery, which is dependent on specific types of drug–plasma protein interactions, constitutes a subject for further investigation.

## Data Availability

The datasets generated during and analyzed during the current study are not publicly available due to restrictions and limitations on curating and maintaining the data in an open repository but are available from the corresponding author on reasonable request.

## Abbreviations

AAG: α1-acid glycoprotein.
ANOVA: Analysis of variance
bpm: Breaths per min
EEG: Electroencephalogram
FF: At the focus
FO: Outside of focus
FUS: Focused ultrasound
GSR: Galvanic skin response
PBS: Phosphate buffered saline
PVA: Polyvinyl alcohol
SSEP: Somatosensory evoked potential
I_sppa_: Spatial-peak pulse-average intensity
I_spta_: Spatial-peak time-average intensity
